# Supplementary material for: Predicting the global mammalian viral sharing network using phylogeography
Source: Nat Commun. 2020 May 8;11:2260. doi: 10.1038/s41467-020-16153-4 (PMC7210981; doi:10.1038/s41467-020-16153-4)
Supplement: Supplementary file 1 — Supplementary Information [file 41467_2020_16153_MOESM1_ESM.pdf]

Supplementary Figures for Albery et al., 2020: Predicting the global mammalian viral sharing network using phylogeography

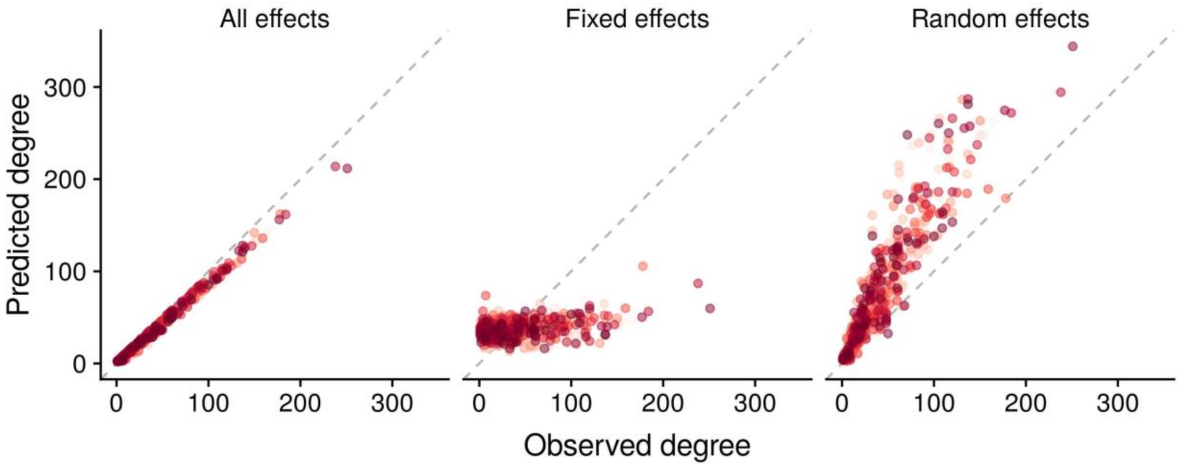

Figure 1: Predicted degree centrality of mammal species in our training data network, predicted using our GAMM estimates. Fixed + random effects were very effective at reproducing individual species' degree centrality (left); fixed effects were less effective (middle); and random effects alone had a strong but imperfect effect (right).

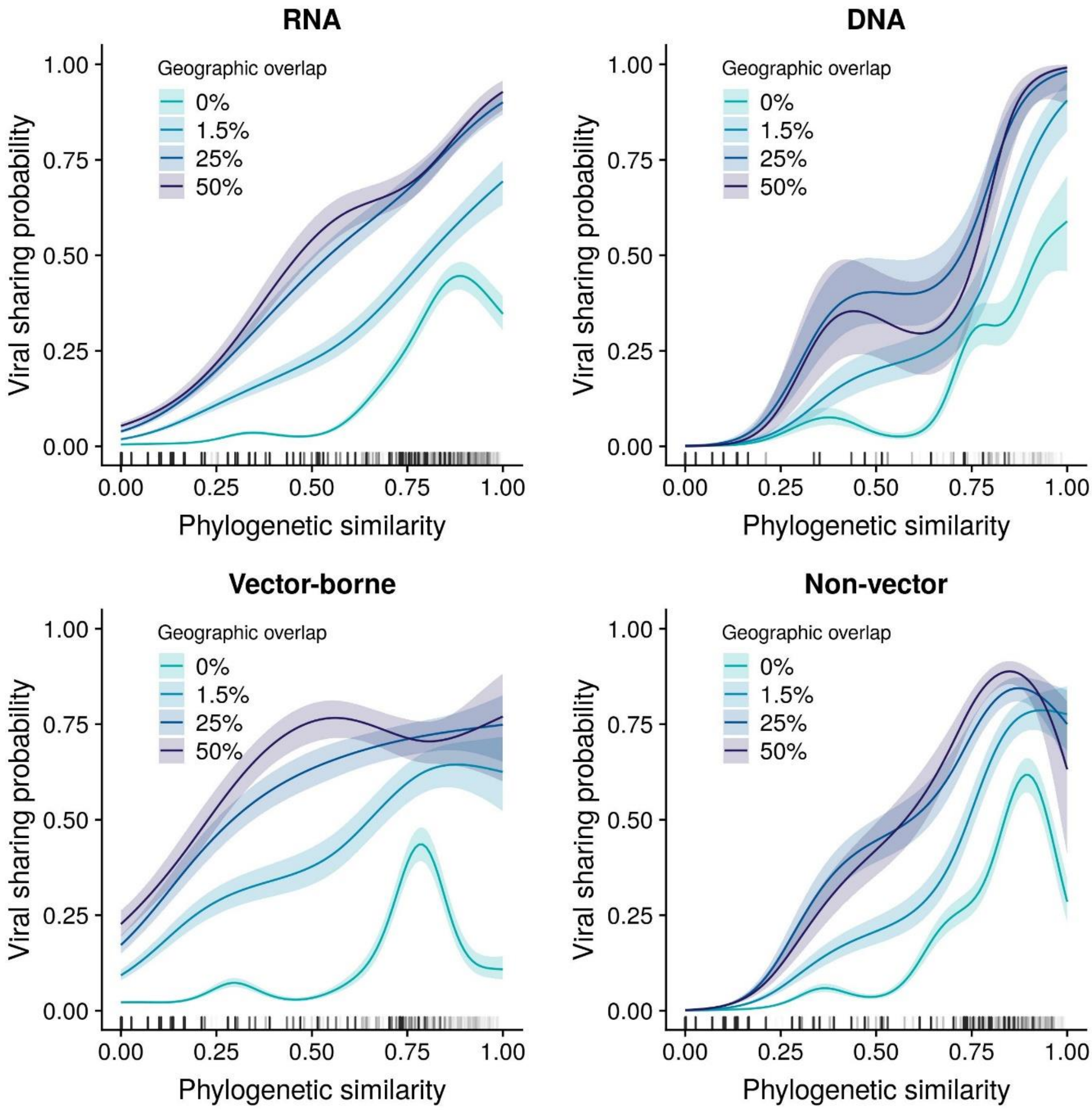

Figure 2: GAMM-derived viral sharing estimates for the effect of phylogenetic similarity for four viral subsets (top row: all RNA viruses and all DNA viruses; bottom row: vector-borne RNA viruses and non-vector-borne RNA viruses). Each GAMM smooth is displayed at multiple geographic overlap values (different colours).

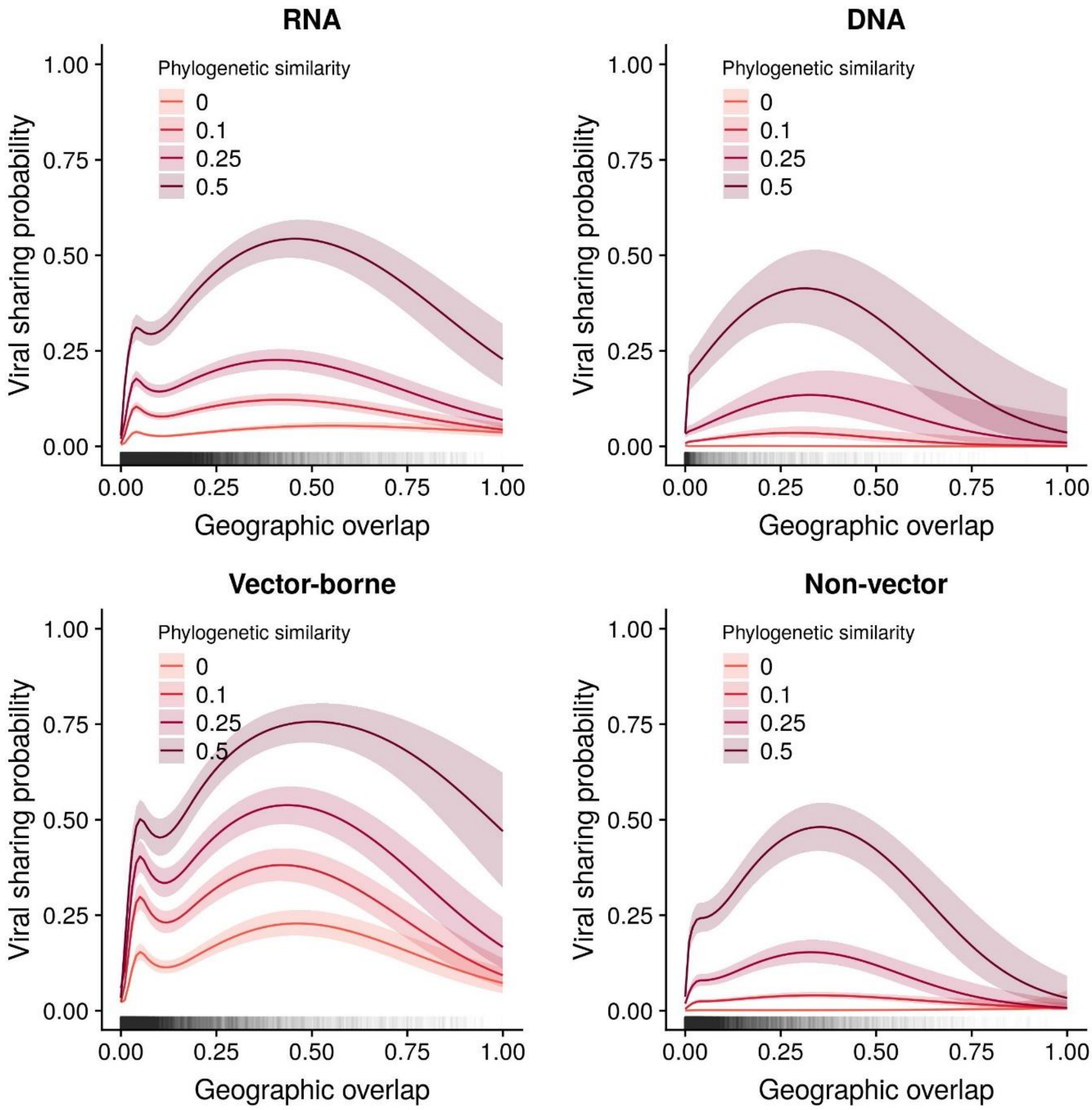

Figure 3: GAMM-derived viral sharing estimates for the effect of geographic overlap for four viral subsets (top row: all RNA viruses and all DNA viruses; bottom row: vector-borne RNA viruses and non-vector-borne RNA viruses). Each GAMM smooth is displayed at multiple phylogenetic similarity values (different colours).

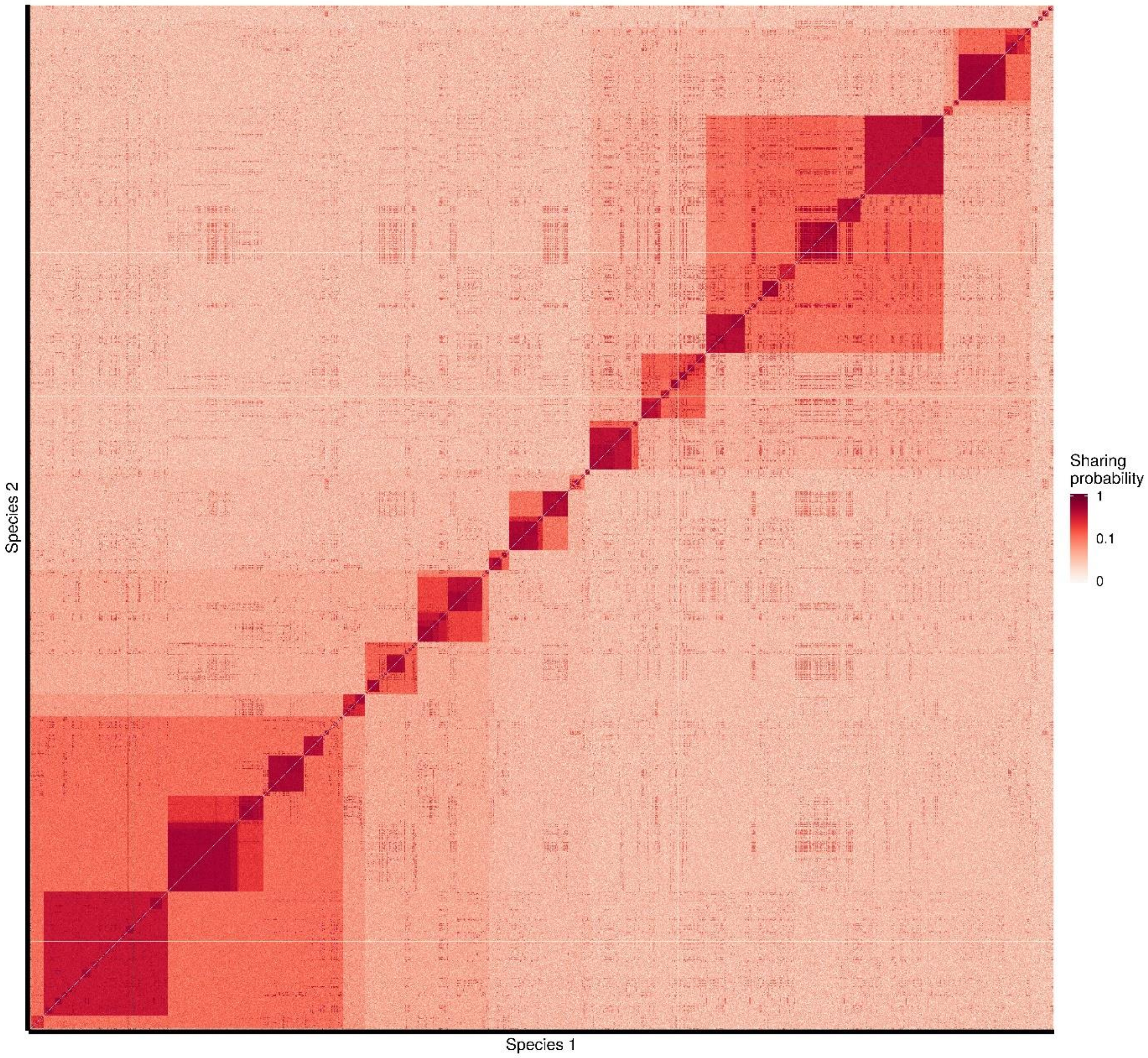

Figure 4: The predicted viral sharing network. Each of the 4196 mammal species in the viral sharing network is represented as a row and a column in the matrix, ordered according to their appearance as a tip in the mammalian supertree. Darker red colours correspond to increased pairwise viral sharing probability between two species; the colour gradient is on a log10-scale to improve interpretability.

RESPONSE	SAMPLES	DEVIANCE CONTRIBUTIONS					
		Geography	Gz	Phylogeny	Citations	Domestic	Spp
**ALL VIRUSES**	591	0.077	0.067	0.336	0.005	0.002	0.512
**RNA**	566	0.079	0.067	0.33	0.005	0.003	0.516
**DNA**	151	0.008	0.031	0.729	0.001	0.004	0.227
**VECTOR-BORNE**	333	0.153	0.11	0.145	0	0.008	0.584
**NON-VECTOR**	391	0.011	0.019	0.625	0.016	0.001	0.328

Supplementary Table 1: The deviance contributions and sample sizes (number of hosts) for each of our viral sharing GAMMs. The deviance terms are, in order: proportional geographic overlap; binary geographic overlap greater than zero (Gz); phylogenetic similarity; minimum citation number; domestication status; and the species-level random effect.

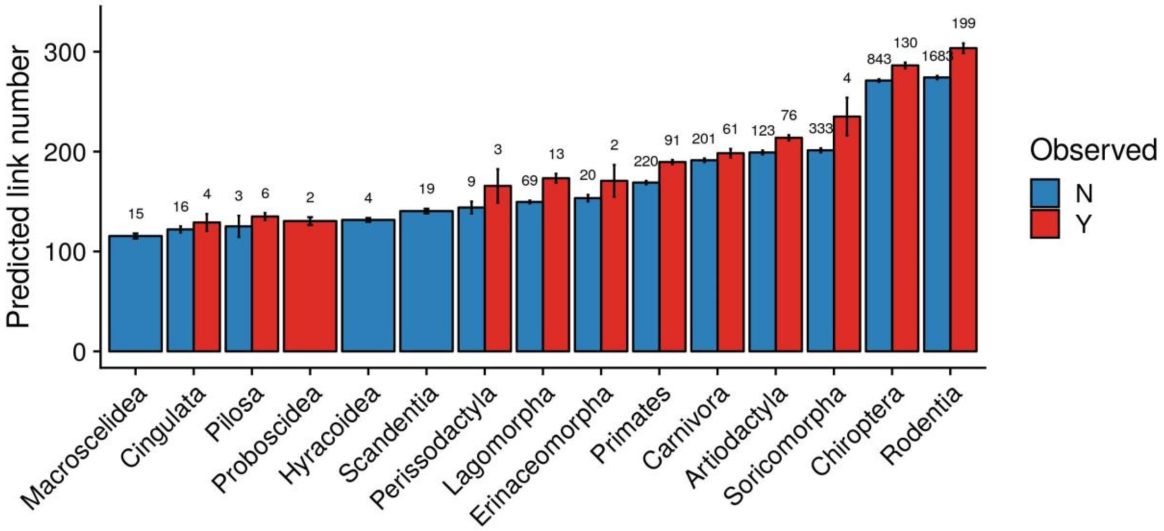

Figure 5: Mammal species that were observed with at least one virus in the training dataset or the EID2 dataset had higher degree centrality (link number) in our predicted network. This figure displays the raw data that are displayed in Figure 2C in the main text, but without being scaled within orders. Bars represent means; error bars represent standard errors.

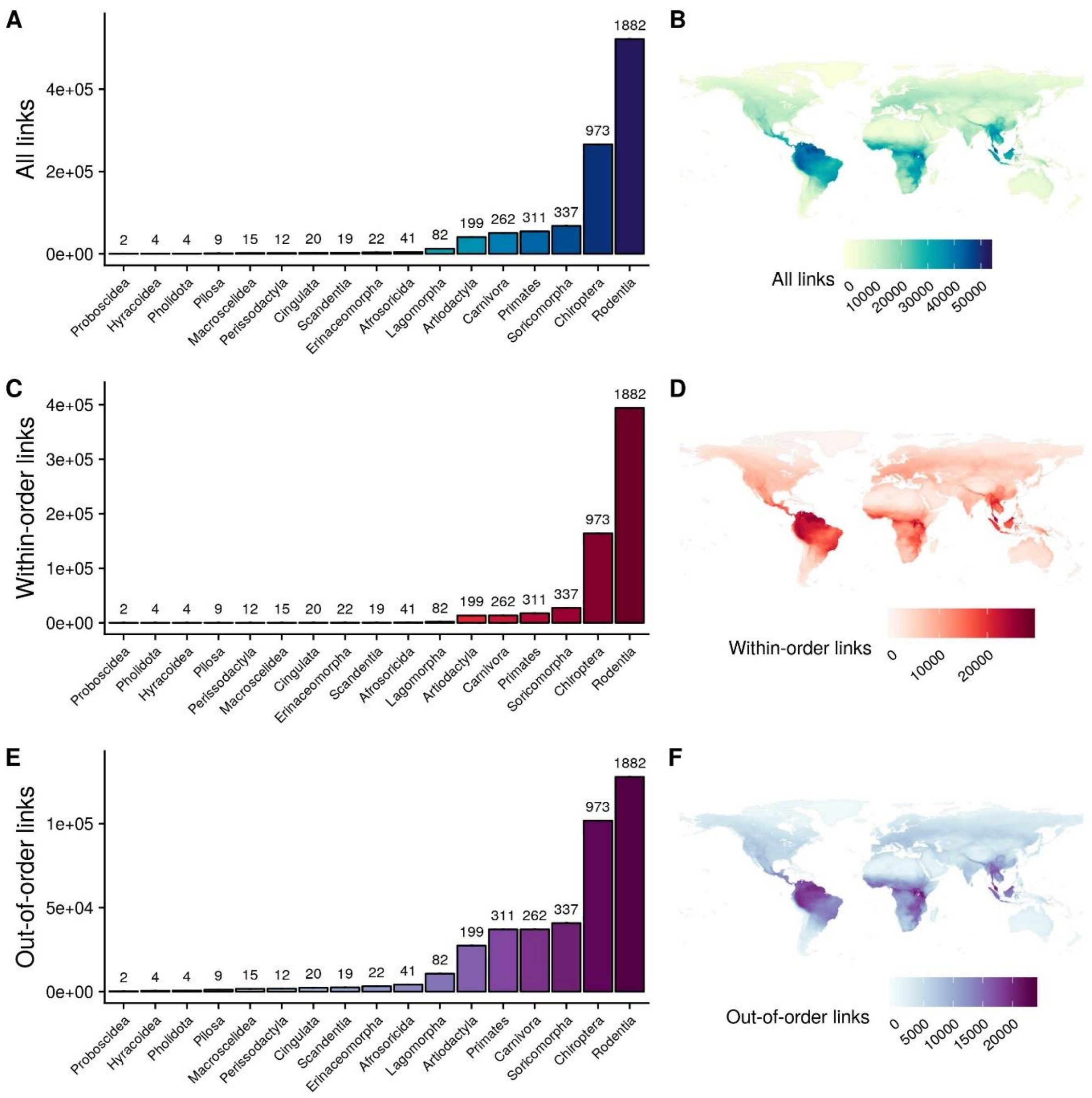

Figure 6: Taxonomic and geographic patterns of predicted viral link numbers. Top row: all links; middle row: links with species in the same order; bottom row: links with species in another order. A,C,E: summed species-level link numbers for mammalian orders in our dataset. Bars represent means; error bars represent standard errors. B,D,F: geographic distributions of link numbers. Distributions were derived by summing the link numbers of all species inhabiting a grid square.

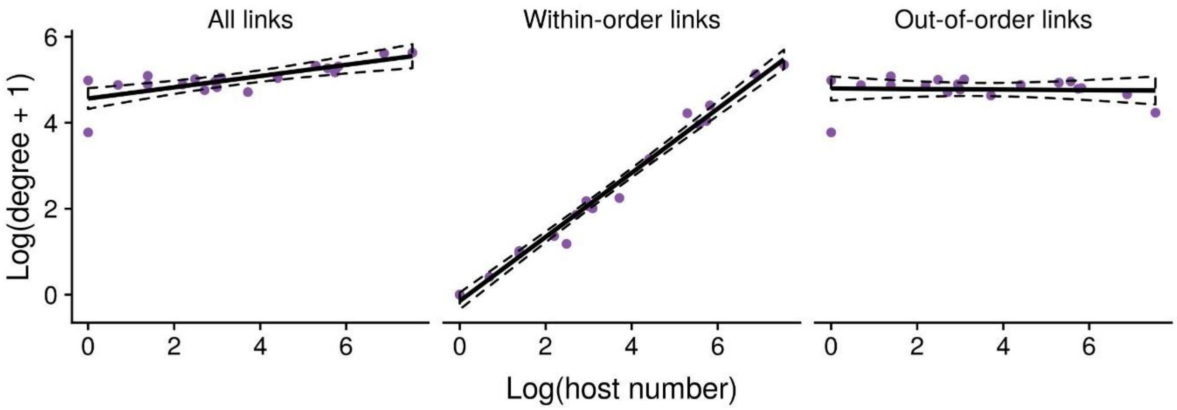

Figure 7: Scaling of degree centrality (link numbers) followed a power law when looking within-orders, but not between orders. The trend line and 95% confidence intervals are derived from a linear model fitted to the data (N=17 orders).

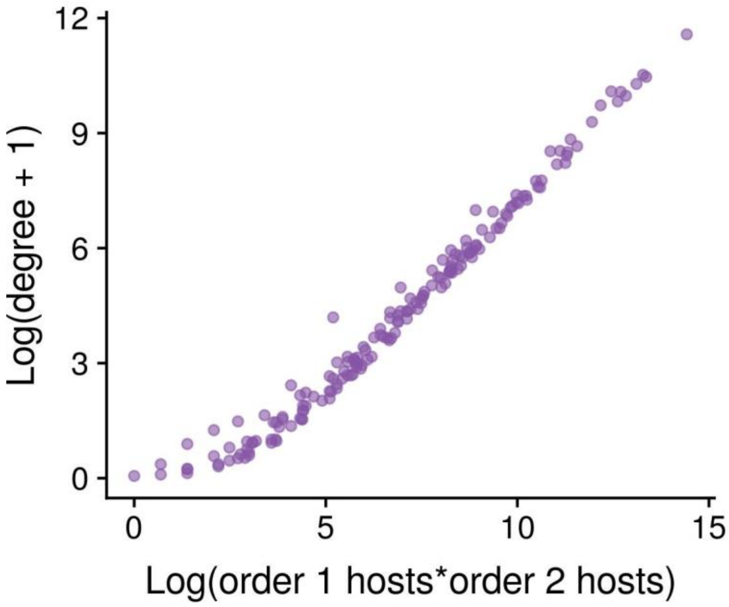

Figure 8: Predicted between-order link numbers scales according to the log-product of the number of species in the two orders. Each point represents a pair of orders (N=171).

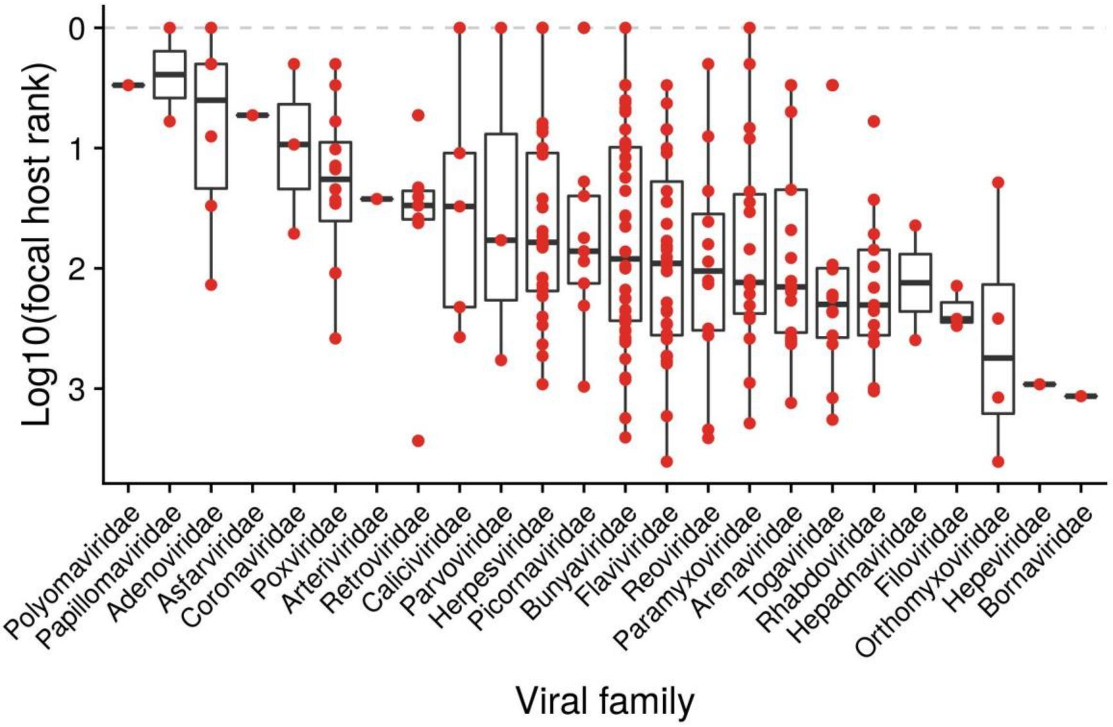

Figure 9: The phylogeographic predictability of viruses' reservoir hosts varied considerably across viral families, although the family-level random effect did not account for much of the model's variance. Families are ordered along the x axis in order of decreasing predictability. The y axis displays the mean rank of the focal host in our reservoir host prediction simulation, on a reversed log10-scale. Values closer to the top of the figure represent viruses with more predictable hosts. The whiskers represent the range of the data, minus outliers; Box plots represent the quartiles, with the median in the centre.

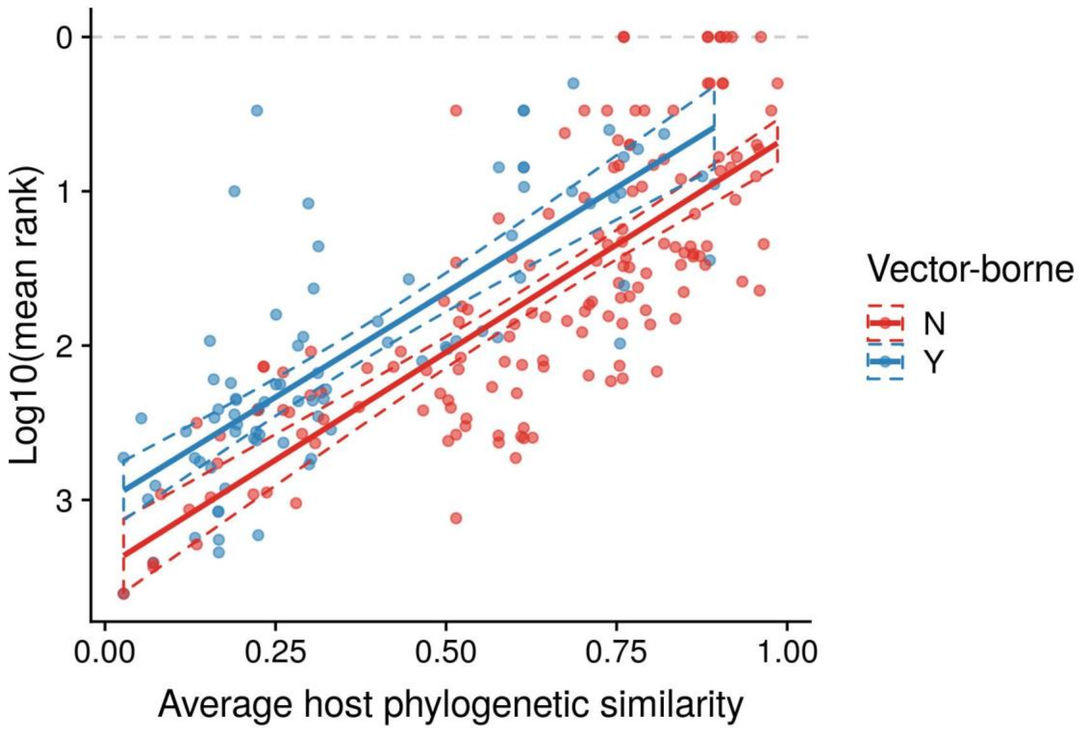

Figure 10: Viral host range strongly impacted the predictability of reservoir hosts. The x axis displays the mean phylogenetic similarity of a virus's hosts (i.e., an inverse measurement of viral host range). The y axis displays the mean rank of the focal host in our reservoir host prediction simulation, on a reversed log10-scale. Each point represents a unique virus; values closer to the top of the figure are viruses with more predictable hosts. The trend lines and 95% confidence intervals were derived from a linear mixed model fitted to the data (N=250).
